# Multivariate sharp‐wave ripples in schizophrenia during awake state

**DOI:** 10.1111/pcn.13702

**Published:** 2024-06-24

**Authors:** Takefumi Ohki, Zenas C. Chao, Yuichi Takei, Yutaka Kato, Masakazu Sunaga, Tomohiro Suto, Minami Tagawa, Masato Fukuda

**Affiliations:** ^1^ International Research Center for Neurointelligence (WPI‐IRCN), The University of Tokyo Institutes for Advanced Study The University of Tokyo Tokyo Japan; ^2^ Department of Psychiatry and Neuroscience Gunma University Graduate School of Medicine Maebashi Japan; ^3^ Tsutsuji Mental Hospital Tatebayashi Japan; ^4^ Gunma Prefectural Psychiatric Medical Center Isesaki Japan

**Keywords:** energy landscape analysis, generalized eigendecomposition, neural oscillations, phase‐amplitude coupling, spontaneous activity

## Abstract

**Aims:**

Schizophrenia (SZ) is a brain disorder characterized by psychotic symptoms and cognitive dysfunction. Recently, irregularities in sharp‐wave ripples (SPW‐Rs) have been reported in SZ. As SPW‐Rs play a critical role in memory, their irregularities can cause psychotic symptoms and cognitive dysfunction in patients with SZ. In this study, we investigated the SPW‐Rs in human SZ.

**Methods:**

We measured whole‐brain activity using magnetoencephalography (MEG) in patients with SZ (*n* = 20) and sex‐ and age‐matched healthy participants (*n* = 20) during open‐eye rest. We identified SPW‐Rs and analyzed their occurrence and time‐frequency traits. Furthermore, we developed a novel multivariate analysis method, termed “ripple‐gedMEG” to extract the global features of SPW‐Rs. We also examined the association between SPW‐Rs and brain state transitions. The outcomes of these analyses were modeled to predict the positive and negative syndrome scale (PANSS) scores of SZ.

**Results:**

We found that SPW‐Rs in the SZ (1) occurred more frequently, (2) the delay of the coupling phase (3) appeared in different brain areas, (4) consisted of a less organized spatiotemporal pattern, and (5) were less involved in brain state transitions. Finally, some of the neural features associated with the SPW‐Rs were found to be PANSS‐positive, a pathological indicator of SZ. These results suggest that widespread but disorganized SPW‐Rs underlies the symptoms of SZ.

**Conclusion:**

We identified irregularities in SPW‐Rs in SZ and confirmed that their alternations were strongly associated with SZ neuropathology. These results suggest a new direction for human SZ research.

Schizophrenia (SZ) is a prevalent psychiatric disorder characterized by psychotic symptoms and cognitive dysfunction. Atypical neural oscillations in the brain have been linked to SZ,^1^, ^2^, ^3^, ^4^, ^5^ and recent research has highlighted the relevance of sharp‐wave ripples (SPW‐Rs), representative of spontaneous neural oscillations at 80–250 Hz, to the pathophysiology of SZ. While most studies on this topic have been conducted in animal models, only a limited number have been carried out in human.^6^, ^7^ In this investigation, we explore SPW‐Rs in human SZ using magnetoencephalography (MEG), a whole‐brain functional imaging system with high‐frequency resolution.

SPW‐Rs have been shown to play a significant role in memory consolidation by upregulating the generation of SPW‐Rs associated with task‐related content (e.g., replay).^8^, ^9^, ^10^, ^11^, ^12^ However, it has been suggested that the functionality of SPW‐Rs spontaneously generated without an explicit task may reflect internal thoughts and generalized memories with the interaction of other neural oscillations such as phase‐amplitude coupling (PAC).^3^, ^4^, ^13^, ^14^, ^15^ This interplay has implications for the pathology of SZ.^16^, ^17^, ^18^ Furthermore, the functionality of SPW‐Rs is not confined to effects on local neural circuits, such as regulation of synapses,^14^, ^19^ but exert a more global effect across the entire brain and inducing activity in other brain regions.^20^, ^21^, ^22^, ^23^ To address these issues, we developed a novel multivariate approach based on generalized eigendecomposition (GED), which we termed ripple‐gedMEG. GED has been demonstrated to be computationally more efficient and to have a superior signal‐to‐noise ratio in the spatial and temporal domains.^24^, ^25^, ^26^ Here, we tailored GED specifically for the analysis of SPW‐Rs.

Our analysis revealed distinctive features of SPW‐Rs in SZ. Specifically, the number of SPW‐R events in the SZ group was significantly larger than that in the control group, and a delay in the timing of PAC (i.e., the coupling phase) was detected. Furthermore, degeneration of the SPW‐Rs network and the brain state transition associated with SPW‐Rs were significantly diminished in SZ. Furthermore, using generalized linear models, we found that these observed degenerations could significantly predict the positive and negative syndrome scale (PANSS) positivity—a standard assessment tool for SZ. Thus, these results suggest that focusing on SPW‐Rs has the potential to lead to the development of new therapeutic strategies for SZ.

## Materials and Methods

### Participants

A total of 20 patients with SZ and 20 healthy controls (HC) were recruited at Gunma University Hospital between September 2014 and February 2020, with the approval of the ethics committee of Gunma University Hospital (UMIN000026411). The sex and age of the two groups were matched (see Table 1), with 10 males and 10 females in each group, and mean ages of 36.5 (SZ) and 36.4 (HC). Participants were given a detailed description of the purpose of the study and provided written informed consent before joining. This study was conducted in accordance with the most recent version of the Declaration of Helsinki. The Structured Clinical Interview for DSM Disorders, Fourth Edition, Axis I, and Axis II disorders was conducted to exclude participants with a diagnosis of SZ and a history of psychiatric disorders from HC. In addition, we used the PANSS and the Japanese Adult Reading Test (JART) to assess the psychopathology of SZ (Table 1).

**Table 1:** Detailed demographics, clinical and medication information of this study

	HC (*n* = 20)		SZ (*n* = 20)	
	M	F	M	F
	10	10	10	10
Sex	Mean (group)	SD (group)	Mean (group)	SD (group)
Age (year)	36.4	4.6	36.5	5.7
Age range (year)	30–49	‐	28–46	‐
JART	110.1	6.55	102.1*	14.3
PANSS Total score	‐	‐	62.3	22.18
Positive symptoms	‐	‐	13.84	5.99
Negative symptoms	‐	‐	17.78	7.26
Medications				
Antidepressant (imipramine equivalent dose mg/day)	‐	‐	5	15.91
Antipsychotic (chlorpromazine equivalent dose mg/day)	‐	‐	585.15	438.8
Anxiolytic (diazepam equivalent dose mg/day)	‐	‐	0.37	1.16
Hypnotic (flunitrazepam equivalent dose mg/day)	‐	‐	0.58	0.84
Antiparkinsonism (biperiden equivalent dose mg/day)	‐	‐	1.05	1.27

HC, healthy control; JART, Japanese Adult Reading Test; M and F, male and female; PANSS, Positive and Negative Syndrome Scale; SD, standard deviation; SZ, schizophrenia.

* *P* < 0.05.

### MEG data acquisition and preprocessing

We measured brain activity in 40 participants during 7 min of open‐eye rest. During measurement, the participants were required to gaze at a fixation point and relax in an upright (sitting) position. The MEG device used in the current study was a 306‐channel Elekta NeuroMag (Oy, Helsinki, Finland) installed in a magnetically shielded room (JFE Mechanical Co., Tokyo, Japan). The sampling rate was set to 1002 Hz. In addition to MEG measurements, electrodes for electrocardiography (ECG) and electrooculography (EOG) were attached to the body and face to measure the effects of biological artifacts, such as heartbeat, blinking, and other eye movement‐related potentials. Careful attention was paid to the SZ's state of arousal, which was constantly monitored by a camera inside the shielded room during imaging, and we confirmed that the SZ's arousal state was adequately maintained. After the measurements, the Stanford Sleepiness Scale was also used to check participants' wakefulness (see the relevant section in Supporting information (SI)).

As preprocessing to remove system noise such as alternating current (AC) and biogenic artifacts, we used Max filter 2.0 (Elekta‐Neuromag), a signal space projection, an independent component analysis (ICA) to remove components with amplitudes >2000 fT, and a notch filter. The sampling rate was then downsampled from 1002 to 1000 Hz. All offline data processing was performed using MATLAB R2021a (MathWorks) and the brainstorm package.^27^ For more detailed preprocessing, please refer to SI.

### Data analysis procedure for SPW‐Rs

#### Auto‐detection of SPW‐Rs

An automatic detection algorithm was used for the timing detection of the SPW‐Rs.^28^ This algorithm requires two parameters: alpha threshold and sampling rate. We set the alpha threshold and sampling rate to 0.01 and 1000 Hz, respectively. It's important to note that we made adjustments to this algorithm: the detection frequency (80–250 Hz) and introducing a parameter for the event duration threshold (10–100 ms) based on physiological validity.^29^, ^30^ The selection and adjustment of this algorithm were determined by a pilot study involving simultaneous measurements with stereoelectroencephalography (sEEG) and MEG. The automatic detection algorithm was applied to each MEG sensor. Consequently, events detected by multiple sensors within the same time bin (10–100 ms) were treated as identical to account for potential signal leakages. For details and issues regarding SPW‐Rs detection using MEG, please refer to the relevant section in SI.

#### Quantification of basic SPW‐Rs characteristics *via* the univariate approach

**Fig. 1:**
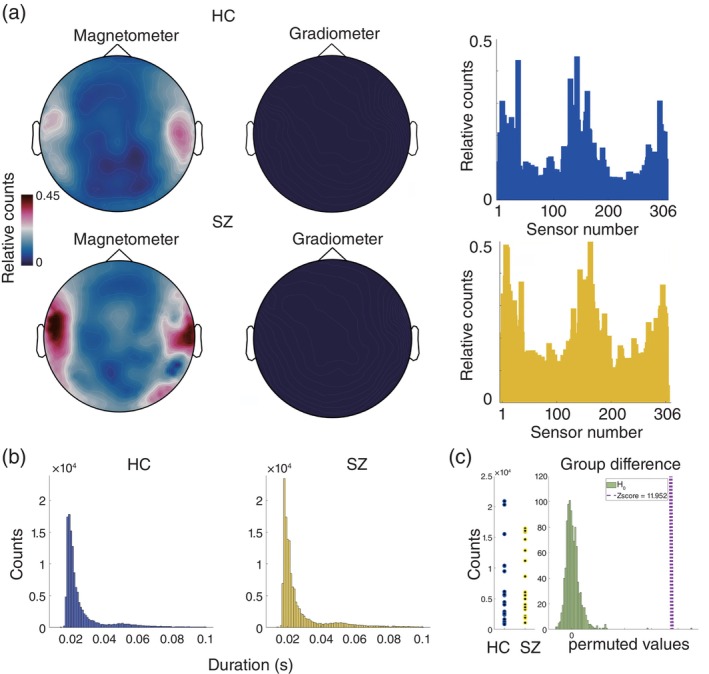
Sharp wave ripples *via* auto‐detection and the basic traits on sensor‐level. (a) The sensor location detecting sharp wave ripples (SPW‐Rs). The upper row represents the healthy group denoted as HC, and the lower row represents the schizophrenia group represented as SZ. The auto‐detection results were separately plotted based on the MEG sensor type: magnetometer left and gradiometer right. In this plot, the number of events was converted to a relative number on a color scale from 0 to 1. In the right panel, this relative number is illustrated as a histogram, with the y‐axis representing the relative number and the x‐axis representing the sensor number. The blue color represents the HC group, and the yellow the clinical group. (b) Histograms of the number and duration of SPW‐Rs. The blue histogram on the left represents the healthy group and the yellow histogram on the right represents the clinical group. The y‐axis represents the number of events and the x‐axis represents the duration of events. (c) The number of events for each participant in two groups and surrogate data distribution. The two left columns illustrate a scatter plot showing the number of events in each participant. The blue and yellow circle denotes the HC group and SZ, respectively. In the right column, the null hypothesis distribution in light green for the number of events is illustrated. The value of this null distribution is z‐valued. The purple vertical dotted line represents the *Z*‐value of the current data (i.e., the difference of counts in HC and SZ).

The following analysis was performed to quantify the univariate SPW‐Rs. First, we examined the spatial distribution of events based on MEG sensor type (Fig. 1a, left and middle) and histograms per MEG sensor (Fig. 1a, right). In this analysis, the number of events for each sensor was converted into relative numbers by dividing the number of events by the maximum number of events for each participant. We showed a histogram of the event duration for each group (Fig. 1b). To investigate the differences in the number and duration of events between the groups, we performed a permutation test (Fig. 1c, SI for detailed procedures).

#### Quantification of time series information of SPW‐Rs

**Fig. 2:**
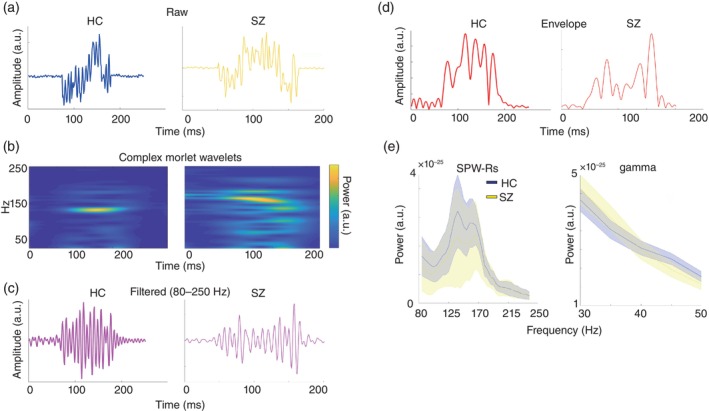
Time‐frequency decomposition of sharp‐wave ripples. (a) A typical sharp‐wave ripple (SPW‐Rs) as the raw waveform detected by the MEG sensor. An example shown in blue indicate SPW‐Rs observed in the HC group (left) and yellow (right) in the schizophrenia group (SZ). X‐axis represents time (ms), and the y‐axis represents amplitude. (b) Time‐frequency analysis with complex morlet wavelets. The left panel shows the HC group and the right panel shows the SZ group. Both SPW‐Rs have a peak around 150 Hz. Amplitudes and envelopes of SPW‐Rs after filtering (80–250 Hz) in (c) and (d). The left panel shows the HC group and the right panel shows the SZ group. (e) Grand‐averaged power of SPW‐Rs and gamma in HC (blue) and SZ (yellow) groups. Power of SPW‐Rs (80–150 Hz) on the left and gamma (30–50 Hz) on the right. The thick blue (HC) and yellow (SZ) lines represent the grand average power of both groups, and the lightly shaded areas represent the standard deviations between the participants.

To reveal the time‐frequency constituents of SPW‐Rs (Fig. 2a), we calculated the time‐series power from the 30 to 250 Hz bands *via* complex Morlet wavelet convolution (CMW,^31^ Fig. 2b), amplitude fluctuation (Fig. 2c), and envelope (Fig. 2d) *via* bandpass filtering. The median gamma (30–50 Hz) and SPW‐Rs power values of all events from each group were obtained using CMW (Fig. 2e, also see SI).

#### Quantification of PAC *via* the Wasserstein modulation index (wMI)

**Fig. 3:**
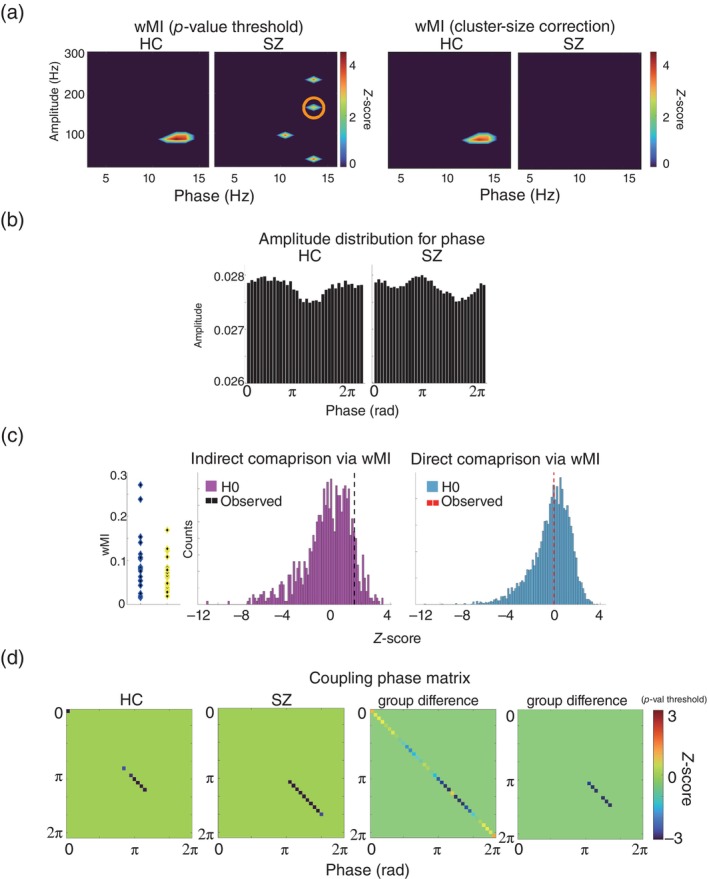
Phase amplitude coupling (PAC) *via* Wasserstein Modulation Index (wMI). (a) The z‐scored comodulograms with the *P*‐value thresholding (left) and cluster size correction (right) were created to reveal the frequency characteristics of the PACs of sharp‐wave ripples (SPW‐Rs) for both groups. The x‐axis and y‐axis denote the phase frequency (Hz) and the amplitude frequency (Hz), respectively. The panels represent the results of the PAC analysis for the healthy control group (HC) and for the schizophrenia group (SZ). The data from the pixels highlighted in the orange circle are utilized in the schematic representation in panels BCD. (b) The group averaged amplitude distribution for phase. The x‐axis represents phase (rad) and the y‐axis represents amplitude modulation (probability). This plot uses pixels that were significant in panel A (13 and 90 Hz for phase and amplitude in HC; 13 and 150 Hz for phase and amplitude in SZ). (c) Group comparison for the group‐averaged coupling strength. The left panel shows the individual raw wMI values in the HC (blue) and SZ group (yellow). The left and right histogram in purple and light blue are the two distinctive surrogate data distributions generated by the indirect and direct approach. The dotted lines denote the observed wMI values in z‐score. (d) The z‐scored coupling phase matrices. From the left to the right, results for HC, SZ and the group comparisons without and without adapted *P*‐value thresholds (*P* < 0.001). The diagonal elements of these matrices denote the significant coupling phase position.

SPW‐Rs exhibit reciprocal interactions with other neural oscillations.^3^, ^15^ A typical example is the PAC.^13^, ^32^, ^33^, ^34^ Therefore, we quantified the PAC for SPW‐Rs in both groups to confirm the authenticity of SPW‐R events (Fig. 3). Two crucial points in quantifying PAC are the coupling intensity and timing (i.e., the coupling phase).^2^, ^35^, ^36^ To address these aspects, we utilized our original method, wMI,^4^ since wMI possesses distinctive computational properties by applying optimal transport as the distance function to the analysis of the amplitude patterns in the phase plane. Notably, wMI excels in precisely quantifying coupling strength and coupling phase, even in cases of limited number of data and short data length, and proves to be particularly valuable in conditional or group comparisons.^4^

In the wMI computation, we first concatenated the event data for each participant and applied a band‐pass filter and Hilbert transformation to extract the low‐frequency phase information with 1 Hz increment from 3 to 16 Hz and the high‐frequency amplitude information with 10 Hz increment from 50 to 300 Hz. The wMI calculations were performed using a combination of filtered low‐ and high‐frequency data (Fig. 3a). The number of phase bins was set to 36 (i.e., one phase bin represented 10°, Fig. 3b).

There are two main outputs of wMI: the wMI value representing the coupling intensity and the coupling matrix representing the coupling phase. For the statistical evaluation of these values, three surrogate data sets were generated (Fig. 3a,c,d) (see SI). After creating the surrogate data, the observed wMI and coupling phase matrix were converted to z‐scores, and *P*‐values were obtained. Additionally, we performed a direct group comparison between the HC and SZ groups, utilizing a computational property of wMI, as shown in Fig. 3c (i.e., direct comparison). For some figures (Fig. 3a,d), a threshold based on *P*‐values (*z* = ± 3.29, *P* < 0.001) and correction for multiple comparisons using cluster‐based statistics (*P* < 0.01) was applied, and values that did not reach the threshold were replaced with 0. Refer to SI for the raw results without thresholding (Fig. S1) and procedures of cluster‐based statistics (cluster‐size correction).

#### Multivariate SPW‐Rs using GED (ripple‐gedMEG)

**Fig. 4:**
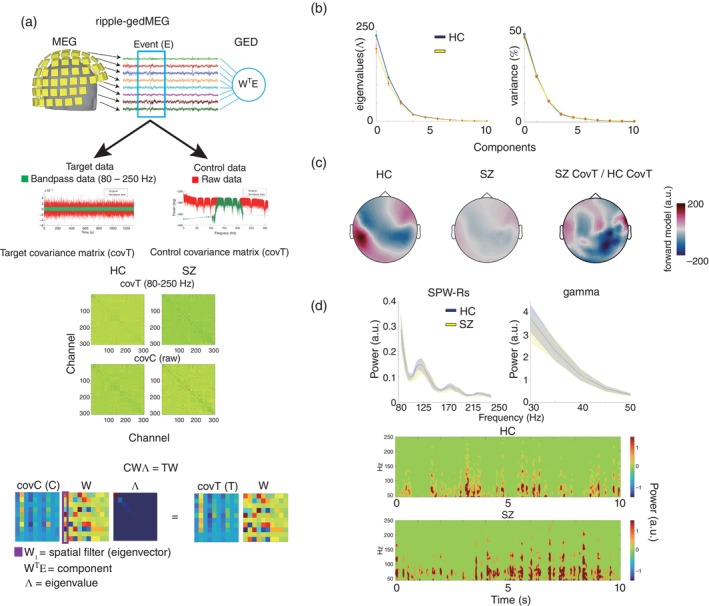
Multivariate sharp wave ripples *via* generalized eigendecomposition (ripple‐gedMEG). (a) Schematic representation of ripple‐gedMEG. The upper panel illustrates the analysis procedure of ripple‐gedMEG. The target event (e) detected with MEG *via* the automatic detection algorithm highlighted by the blue box was applied to generalized eigendecomposition (GED). The middle panel illustrates the creation of the two covariance matrices as the core parts of the GED. First, for the target data, a bandpass filter (80–250 Hz) was applied to extract the neural features associated with the target event (i.e., SPW‐Rs). On the other hand, for the control data, the raw data was used. Using these target and control data, two covariance matrices consisting of channel × channel were created for each; that is, covT and covC in the HC and SZ group. In the lower part of the figure, the mathematical description for ripple‐gedMEG (CWΛ=TW) and the three mathematical outcomes are described; *W*_1_ (a spatial filter or eigenvector associated with the ripple network), *W* ^T^ *E* (component, time‐series activity pattern of the ripple network), and Λ (eigenvalue, the strength of the ripple network). (b) Eigenvalues and variance (%). In these panels, two eigenvalues (left) and variance of ripple‐gedMEG (right) for the HC (blue) and SZ group (yellow) were depicted. The y‐axis shows eigenvalues and variance (%), and the x‐axis represents the components. (c) The forward models *via* ripple‐gedMEG. This figure depicts the whole‐brain network pattern composed by SPW‐Rs. From left to right, the forward models for the HC and SZ groups are shown. The rightmost panel shows the spatial filter that directly compared the differences between the ripple network patterns of the HC and SZ groups. In this calculation, the SZ covT and the HC covT shown in (a) were used (i.e., SZ covT/HC covT). (d) Comparison of frequency power of components. The thick blue and yellow line represents the grand averaged values of the HC group and SZ, respectively. The shaded areas represent standard deviations of each group. The bottom panel shows an example of the time‐frequency analysis of the component during 10 s.

The primary strength of the current study was the quantification of SPW‐Rs using whole‐brain measurements with MEG. To fully leverage this advantage, we used a novel multivariate approach to analyze SPW‐Rs, which allowed us to reveal the whole brain network consisting of SPW‐Rs. For this purpose, we applied the GED. The primary objective of applying the GED was to separate the brain network consisting of SPW‐Rs from other brain activities.^24^, ^37^, ^38^ To implement this, we initially created two covariance matrices: the target covariance matrix denoted as covT or T (channel‐by‐channel) associated with SPW‐Rs and the control covariance matrix consisting of the raw brain signals denoted as covC or C (channel‐by‐channel). As shown in Fig. 4a, the T matrix incorporates the spatial feature of SPW‐Rs (i.e., 80–250 Hz), while the C matrix represents the one of the raw signals. These two matrices were used to perform eigenvalue decomposition. Specifically, GED can be expressed as

CWΛ=TW

or

$$\mathrm{W\Lambda}=\left(C^{-1}T\right)W$$

Here, W denotes the channel weights or spatial patterns known as eigenvectors, and Λ represents a signal‐to‐noise ratio (SNR) for the signal source separation called as eigenvalues. Thus, the GED aims to find spatial patterns (i.e., W) that differentiates covT from covC, and Λ serves an index of SNR for the source separation. Note that GED ignores common spatial patterns between T and C, and W on the left‐hand side and right‐hand side in the upper formula are identical. For *C*, shrinkage regularization (SR) was applied for regularization in order to reduce overfitting and increase numerical stability (see Fig. S2).

We obtained three main outputs *via* ripple‐gedMEG. Firstly, ripple‐gedMEG provides eigenvalues (Λ), signifying SNR for the source separation (Fig. 4b). In this study, we used eigenvalues as the indices signifying the strength of the SPW‐Rs network. In addition, the explanatory power of the ripple‐gedMEG was calculated by converting the eigenvalues to percentages (%) (Fig. 4b). Second, a set of spatial weights denoted as W (i.e., spatial filter) that represents the spatial patterns of SPW‐Rs was produced. This spatial filter (W), when multiplied by T, is referred to as the forward model ($W^{T}$), providing a more direct representation of the SPW‐Rs networks (Fig. 4c). Third, the transposition of the spatial filter ($W^{T}$) with multiplication by all the sensor time‐series information is the time‐series data of the SPW‐R network, known as a component ($W^{T}E$). We applied CMW to the components (Fig. 4d). Note that we only focused on the first eigenvector ($W_{1}$), eigenvalue, and component that are most directly SPW‐Rs. As a statistical process in ripple‐gedMEG, we used the permutation test and Wilcoxon rank‐sum test (see SI in detail). Notably, the superiority and robustness of ripple‐gedMEG using the simulation data are demonstrated in Figs. S3–S6.

#### Source estimation *via* linearly‐constrained minimum variance (LCMV) beamformer

To map the MEG source‐estimated data to the normalized brain coordinate system (i.e., MNI coordinates), we acquired structural MRI T1‐weighted images from all participants using a 3T MRI scanner (Philips Medical Systems, Netherlands) with a 12‐channel phase‐array receiver coil (see SI for setting the MRI parameters). For the T1 image data, we applied the FreeSurfer^39^ to reconstruct the cortical maps and coalign the individual cortical maps and MNI coordinates. Then, the original number of vertices was downsampled to 15,002 vertices, using the Brainstorm toolbox.^27^ Second, we used a linearly constrained minimum variance (LCMV) beamformer and performed source estimations. The LCMV beamformer, an adaptive distributed‐source imaging method, utilizes not only sensor location information but also spatial weights (covariances or cross‐spectral densities) derived from data characteristics. This enables the detection of low‐amplitude brain activities with a coherent spatial distribution, especially in multi‐sensor systems like MEG. However, it involves a more extensive parameter settings for computation compared to non‐adaptive distributed‐source imaging methods, such as the minimum norm estimate. For this study, resting‐state data and 5‐min empty room data were used to calculate the data and noise covariance matrices. The median eigenvalue was employed for regularization of the data covariance. Regarding dipole orientations, the unconstrained option was selected.

#### Energy Landscape Analysis (ELA) for elucidating the brain state transitions

**Fig. 5:**
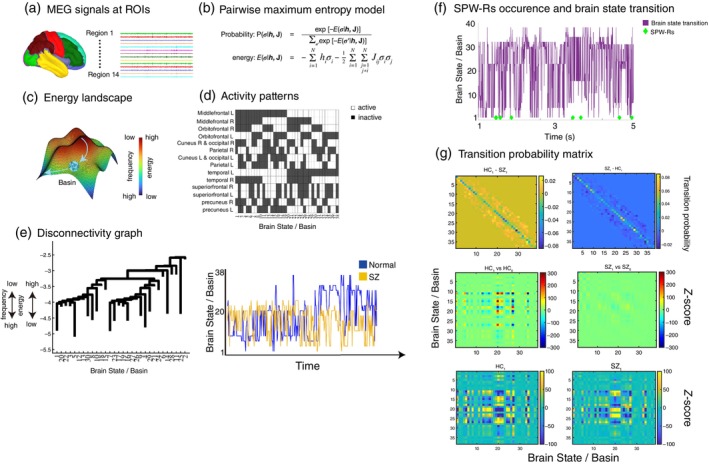
Effects of SPW‐Rs on the transition probability of the brain states. (a) The source estimated data for the whole brain *via* the beamformer were extracted by principal component analysis based on 14 regions of interests (ROIs). These sources estimated data were used for Energetic Landscape Analysis (ELA). (b) The pairwise maximum entropy model (MEM). The MEM model was fitted to all activations of 14 ROIs ($2^{14}$), and the energy value was calculated for each activity pattern. (c) Schematic illustration of ELA. Each local minimum corresponds to the bottom of the basin and is equivalent to the brain state. (d) The activity patterns of 14 ROIs corresponding to the bottom of the basin were depicted. A white and black cell denote an active and inactive respectively. (e) Relationships between the brain states (i.e., the basins) were quantified as the disconnectivity graph. The right panel shows another important output of the ELA, the brain state time series (blue and yellow represent HC and SZ, respectively). (f) Appearance of SPW‐Rs and the transitions of the brain states. The purple line indicates the time‐series of the brain state transition. The green dot at the bottom represents the time when SPW‐Rs were generated. (g) The brain state transition matrices associated with SPW‐Rs. The x‐axis and y‐axis represent the brain states, respectively. The upper panel shows the difference of transition probability between HC and SZ in the time window when SPW‐Rs were generated. The middle panel shows significant differences in the state transitions between in the time window with SPW‐Rs (denoted as HC_1_ and SZ_1_) and in the time window without SPW‐Rs (denoted as HC_0_ and SZ_0_). In the bottom panel, the group differences between time windows with SPW‐R (HC_1_ and SZ_1_).

In this study, ELA^40^ was employed to explore brain state transitions associated with occurrence of SPW‐Rs^22^, ^41^, ^42^ at the whole‐brain level. ELA interprets state transitions as the movement of a ball within an energetic gradient called energy landscape, estimated from the data (Fig. 5). The advantage of ELA lies in its ability to quantify sequential state transition patterns, based on the original data's sampling rate without relying on sliding time windows and pre‐specifying the number of states. In our ELA calculation, spatially representative time‐series data were extracted using principal component analysis (PCA) for each of the 14 regions of interest (ROIs) based on the Mindboggle‐101 atlas^43^ (Fig. 5a). Due to the computational complexity increasing exponentially with the number of brain regions, the entire brain was divided into 14 regions for ELA calculation in this study.

ELA was then applied to the whole‐brain time‐series data (Fig. 5b–e). To align the brain states (i.e., a “basin” shown in Fig. 5c) between HC and SZ, we concatenated two spatially representative time‐series data sets from both groups. Transition probabilities for brain state changes were subsequently calculated for time‐series data with SPW‐Rs (SPW‐Rs = 1) and without SPW‐Rs (SPW‐Rs = 0) separately (Fig. 5f). To assess the statistical significance of the transition probabilities associated with SPW‐R occurrence and group differences (Fig. 5g), permutation tests were used (see SI for more details).

#### Regression and correlation analysis

**Fig. 6:**
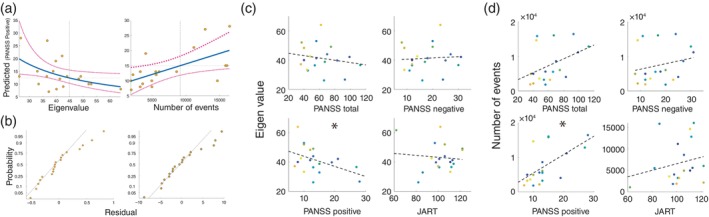
Two generalized linear models (GLM). (a) For these regression models, PANSS and JART for dependent variables, and neural features such as eigenvalues and the number of events for independent values were used. The left and right panel shows the results of GLMs for PANSS positive with eigenvalues and the number of events as the independent variable respectively. The blue line represents the predicted regression line, and the magenta dotted line represents the 95% confidence interval. Each dark orange dot represents data for schizophrenic patients. (b) Residual probability plot. To demonstrate the predicted and residual values, the y‐axis represents data probability and the x‐axis represents residuals. The left panel shows the residual probability plot with eigenvalues and the right panel shows the residual probability plot with the number of events as the independent variable. (c and d) Correlation maps. Scatter plots of eigenvalues (c) and the number of events (d) as the y‐axis, and PANSS total (upper left), PANSS negative (upper right) and PANSS positive (left bottom), and JART (right bottom) as the x‐axis. Each color of the dots in panels c and d represents the same individual SZ data. The dotted lines denote the least‐squares line. The blue asterisks indicate significant correlations.

To examine the potential association between the neural features of SPW‐Rs and the pathology of SZ, we created two generalized linear models (GLM) (Fig. 6a,b) and correlation analysis (Fig. 6c,d): the first GLM using the neural features of SPW‐Rs obtained the univariate analysis and the second GLM *via* the multivariate analysis (i.e., ripple‐gedMEG). The neural features obtained *via* univariate analysis were the frequency power of the SPW‐Rs and the number of events and were used as independent variables. In the creation of the second GLM, the features obtained from the ripple‐gedMEG, such as the eigenvalue and frequency power of the components, were used as independent variables. PANSS: positive, negative, JART and drug treatment obtained from SZ as dependent variables were used for dependent variables (Table 1). We confirmed that these indices were not covariates *via* the partial correlation analysis for both models. To select the model, we initially created a full model encompassing the mentioned independent variables. Subsequently, we systematically removed main effects and interactions of the independent variables, and adopted a model that differed significantly from the previous model. Because behavioral indicators could not be obtained from one patient with SZ in our experiment, GLMs and correlation analyses were performed with the data of 19 patients with SZ. Notably, no significant effects of the drug treatment were detected in our model selection.

## Results

### Quantification of SPW‐Rs characteristics *via* the univariate approach

#### Auto‐detection of SPW‐Rs

In total, 144,092 and 130,542 events were detected in SZ and HC, respectively. Fig. 1a demonstrates the sensor locations that detected the majority of events were localized around the bilateral temporal sensors in both groups, whereas a more widespread spatial distribution pattern unique to SZ. The sensor that detected the events was a magnetometer, and no events were detected in the gradiometers. Fig. 1b shows the histograms of event duration. The median duration (seconds) of all events and variance across participants in HC were 0.0212 and 0.000224 s, respectively, while in SZ, they were 0.0213 and 0.000249 s, respectively. There was no significant difference in the duration of the events (Fig. 1b, permutation test, *P* > 0.05). The total and median numbers of events were 130,542 and 4246 for the HC group and 144,092 and 5261 for the SZ group, respectively. In contrast, the difference in the number of events between the two groups was significant, indicating that more events were observed in the SZ group (Fig. 1b right, permutation test, *P* < 0.001).

#### Quantification of time series of SPW‐Rs

Figure 2a shows the raw temporal waveforms of two typical events in HC (blue) and SZ (yellow). The time‐frequency decomposition *via* CMW results clearly demonstrated high‐frequency oscillatory components around 120–170 Hz in both examples (Fig. 2b). In panels c and d, the amplitude and envelope fluctuations extracted *via* bandpass filtering are shown. We investigated significant differences in the time‐frequency power of all SPW‐Rs and gamma oscillations between the HC and SZ groups (Fig. 2e); however, no significant differences were detected (permutation test, *P* > 0.05).

#### PAC *via* wMI

We observed significant PACs centered at a 10–13 Hz phase and amplitude at 100–250 Hz in both groups (permutation test, *z* = ± 3.29, *P* < 0.001, Fig. 3a). A more focal coupling pattern was observed in HC (left panel). In contrast, in SZ, the significant clusters tended to break into four smaller clusters, as shown in the right panel. When applying the cluster size correction, no significant PAC was detected in SZ (*P* > 0.01, Fig. 3a right). We conducted a group comparison for four clusters, and we report the results from one cluster highlighted by orange circles that were closest to the coupling pattern in HC (13 Hz phase and 160 Hz). Next, we checked the group averages of the amplitude distribution for the phase in the significant clusters to confirm group differences in the coupling intensity and coupling phase. The statistical evaluations (i.e., the indirect and direct comparison) showed no difference in the coupling intensity (Fig. 3c), but a significant difference in the coupling phase (permutation tests, *z* = ± 3.29, *P* < 0.001, Fig. 3d). In other words, the PAC of SPW‐Rs in SZ was significantly delayed compared to that of HC.

#### Ripple‐gedMEG

We observed a significant eigenvalue difference between HC and SZ (Wilcoxon rank‐sum test, *P* < 0.001). In contrast, the similarity in the SPW‐R networks between the two groups was significant (rho = 0.6936, *P* < 0.001). These findings suggest that the SZ group had a similar SPW‐Rs network pattern to HC, but the network formation was weaker than that of HC (Fig. 4b,c). Forward models of the SPW‐Rs are shown in the right panel of Fig. 4c. The grand averaged power of the components obtained from all events *via* ripple‐gedMEG is depicted in Fig. 3d. A clear peak in power was detected in both groups at 120–180 Hz, no group differences were found in either gamma power or SPW‐R (permutation test, *P* > 0.05).

#### ELA for elucidating the brain state transitions associated with SPW‐Rs

To clarify the correspondence between SPW‐Rs and brain state transitions, we performed ELA on source‐estimated time‐series data (Fig. 5a–c). As results, we found that the source estimated time‐series data based on 14 ROIs were successfully modeled as 38 activation patterns (r = 0.997, Fig. 5d). Note that r ranges between 0 and 1 for the maximum‐likelihood estimator; when this model produces a distribution closer to the target or empirical data distribution, r becomes closer to 1. The left panel (Fig. 5e) shows the disconnectivity graph, which signifies the relationship between activity patterns. In this graph, the y‐axis represents the frequency (or energy) of the appearance of activity patterns, with lower positions corresponding to higher frequencies. The x axis represents the number of brain states. In the right panel of Fig. 5e,f, we depict the time series of brain state transitions and the occurrence of SPW‐Rs. To investigate whether SPW‐Rs were involved in brain state transitions, transition probability matrices were created (Fig. 5g). The results suggest that occurrence of SPW‐Rs were linked to multiple brain state transitions in HC (permutation test, *z* = ± 3.29, *P* < 0.001). In contrast, in SZ, brain state changes related to SPW‐Rs were less likely to occur, indicating the tendency to remain in the original state. These findings were confirmed by direct group comparisons (permutation test, *z* = ± 3.29, *P* < 0.001).

#### Regression and correlation analysis

To investigate whether the neural features obtained from the above analyses were related to SZ, we performed GLMs and correlation analyses. As a result, the first GLM with the number of events as the independent variable was adopted and found to be statistically significant in predicting PANSS positive for SZ (*t* = −3.35, *P* < 0.01, Fig. 6a, right). In contrast, the second GLM with only eigenvalues as independent variables was adopted and found to significantly predict PANSS positivity (*t* = 2.3004, *P* < 0.05, Fig. 6a, left). Panel b shows the residuals for each individual in the GLMs. On the left are the residuals for the GLM with eigenvalues and on the right are the residuals for the GLM with the number of events. Spearman's correlation was calculated to confirm these results, and significant correlations were found between the number of events and PANSS positivity (Fig. 6d, rho = 0.609, *P* < 0.01), as well as a marginal effect between eigenvalues and PANSS positivity (Fig. 6c, rho = −0.448, *P* = 0.0544).

## Discussion

Recent studies highlight the close association between SPW‐Rs and the pathophysiology of SZ, suggesting the high potential utility of SPW‐Rs for SZ.^6^, ^7^, ^16^, ^44^ In this section, we delve into the study's findings, explore its limitations, and discuss the neural mechanisms underlying the association between SPW‐Rs and SZ. First, the number of events found in the SZ group was significantly larger than that in the HC group (Fig. 1b). The GLM using the number of events as the independent variable successfully predicted PANSS positive scores (Fig. 6a), indicating that the SZ patients who generated more SPW‐Rs tended to show more positive symptoms, such as hallucinations and delusions. Additionally, we observed a positive correlation between the number of events and PANSS positivity (Fig. 6d). In prior research, two neural factors contributing to the augmentation of SPW‐Rs have been reported: dopamine and N‐methyl‐D‐aspartate (NMDA) receptors. Dopamine is a powerful neuromodulator for SPW‐Rs.^45^, ^46^ For example, the hippocampus, a generator of SPW‐Rs, receives strong dopamine inputs from the ventral tegmental area.^47^ Previous study has verified that the generation of SPW‐Rs was upregulated by dopamine *in vitro*.^45^ Importantly, dopamine‐induced enhancement of SPW‐Rs was linked to the emergence of a new repertoire. Namely, dopamine promoted a new formation of synchronous neuronal firing patterns consisting of SPW‐Rs. Importantly, dopamine is considered a contributing factor implicated in the pathophysiology of SZ,^48^ suggesting SZ may arise from excessive dopamine release (dopamine hypothesis). While it remains challenging to noninvasively measure the effects of dopamine on SPW‐Rs in SZ patients, our experiment effectively replicated the overgenerativity of SPW‐Rs observed in previous studies.^18^

Another crucial neural mechanism for the upregulation of SPW‐Rs is the dysfunction of NMDA receptors (glutamate hypothesis), which holds significance in understanding the pathology of SZ.^49^, ^50^ An association between the over‐productivity of SPW‐Rs and NMDA receptors was reported in a knockout mouse lacking hippocampal NMDA receptors.^18^ This knockout mouse showed upregulated SPW‐Rs in a different form than that in the dopamine model. For instance, hippocampal neurons encoding distinctive spatial information that should be differentiated during SPW‐Rs were simultaneously involved in a neural reactivation called replay. A similar trend was recently reported for human SZ.^7^ Thus, our findings suggest that the upregulation of SPW‐Rs serve as a critical neural mechanism in SZ.

Third, we calculated PAC for the SPW‐Rs. We observed a statistically significant PAC consisting of a phase at 10–15 Hz (corresponding to the frequency range of the spindle) and an amplitude at 100–200 Hz in both groups. Consistent with this finding, previous studies have reported that SPW‐Rs robustly exhibit PAC with spindle and slow oscillations.^3^, ^13^, ^51^, ^52^ Our original method yielded several key physiological features of SZ. First, clusters in the comodulogram demonstrated in Fig. 3a tended to be more fragmented in SZ, while there were no significant changes in the coupling strength between groups (Fig. 3b). More importantly, we detected a significant delay in the coupling phase in SZ, another crucial parameter of PAC.^2^, ^35^, ^36^ Notably, PAC is implemented with an adequate excitation/inhibitory balance (E/I balance).^15^, ^53^ Evidence from GWAS and Copy Number Variations of SZ have indicated that the E/I balance is a crucial factor in understanding neuropathology in SZ.^54^, ^55^ Accordingly, our novel findings regarding the delay in coupling phase can be associated with a disturbance of the E/I balance in SZ.

Another novel finding obtained from the current study was alterations in the SPW‐Rs networks in SZ (Fig. 4). While the SPW‐Rs network pattern in the SZ group was significantly similar to that in the HC group, the eigenvalues reflecting the strength of network formation were significantly lower (Fig. 4b,c). These results suggest that the whole‐brain SPW‐Rs network was attenuated in SZ, consistent with the widespread SPW‐Rs occurrence in SZ (Fig. 1a). These findings suggest that local upregulation of SPW‐Rs in SZ does not necessarily guarantee an adequate spatiotemporal structure of the SPW‐Rs network. Importantly, this spatial formation across the entire brain can be a key neural mechanism to elucidate the neuropathology of SZ, since the alteration of SPW‐Rs (i.e., eigenvalues) significantly predicted PANSS scores (Fig. 6a). In association with the findings of the altered SPW‐R network, the broader influence of SPW‐Rs is currently of great significance to the neuroscience community.^20^, ^21^, ^22^, ^56^, ^57^ Norman *et al*. demonstrated a complex and extensive interaction between SPW‐Rs and canonical resting‐state networks.^22^ Consistent with these previous findings, we found that the occurrence of SPW‐Rs in the HC group was strongly associated with certain brain state transitions (Fig. 5g). In contrast, excessive occurrence of SPW‐Rs in the SZ group was not accompanied by brain state transitions (Fig. 5g). Unfortunately, little is known about the global effects of SPW‐Rs in SZ. However, our findings suggest that understanding the global effects of SPW‐Rs is crucial for advancing both the basic research and the understanding of SZ. For future studies, we recommend a more comprehensive validation approach incorporating longer measurement times, such as Non‐Rapid Eye Movement sleep, and larger sample sizes to fully elucidate the characteristics of SPW‐Rs in SZ.

## Conclusion

We revealed irregularities of SPW‐Rs in SZ *via* a univariate and novel multivariate approach and confirmed that alterations in SPW‐Rs, such as upregulation and formation of the SPW‐R network, were associated with the neuropathology of SZ. These findings suggest that SPW‐Rs reflect the brain pathology of SZ.

## Disclosure statement

Masato Fukuda is an Editorial Board member of *Psychiatry and Clinical Neurosciences* and a co‐author of this article. To minimize bias, this co‐author was excluded from all editorial decision‐making related to the acceptance of this article for publication.

## Author contributions

M.T., M.F., and Y.T. designed the study and M.T., Y.T., Y.T., and T.O. collected the data. T.O. analyzed the data, created all figures, and wrote the draft of the manuscript. C.Z., Y.K., M.T., T.S., and M.F. checked the manuscript. All the authors approved the submitted version.

## Supporting information


**Data S1.** Supporting information.
