# Supplementary material for: Multivariate sharp‐wave ripples in schizophrenia during awake state
Source: Psychiatry Clin Neurosci. 2024 Jun 24;78(9):507–16. doi: 10.1111/pcn.13702 (PMC11488617; doi:10.1111/pcn.13702)
Supplement: Supplementary file 1 — Data S1. Supporting information. [file PCN-78-507-s001.docx]

**Supporting information (SI)**

**Multivariate sharp-wave ripples in schizophrenia during awake state**

Takefumi Ohki, PhD, Zenas C. Chao, PhD, Yuichi Takei, MD, PhD, Yutaka Kato, MD, PhD, Masakazu Sunaga, MD, Tomohiro Suto, MD, PhD, Minami Tagawa, MD, PhD, Masato Fukuda, MD, PhD

Due to journal word limitations, additional detailed descriptions that were omitted in the main text are provided below.

**Magnetoencephalography data acquisition**

We measured brain activity in 40 participants during 7 min of open-eye rest. During measurement, the participants were required to gaze at a fixation point and relax in an upright (sitting) position. The MEG device used in the current study was a 306-channel Elekta NeuroMag (Oy, Helsinki, Finland) installed in a magnetically shielded room (JFE Mechanical Co., Tokyo, Japan). Related to acquisitions of three reference points (i.e., nose, left, and right pinna points) to the headframe coordinate system and the head surface position points (approximately 150-200 points), we used the Isotrak 3D digitizer (Polhemus, Colchester, Vermont, USA). Prior to the MEG data collection, each participant was adjusted with a pillow, cushion, or blanket to ensure proper posture during imaging.

The sampling rate was originally set to 1002 Hz. In addition to MEG measurements, electrodes for electrocardiography (ECG) and electrooculography (EOG) were attached to the body and face to measure the effects of biological artifacts, such as heartbeat, blinking, and other eye movement-related potentials. Careful attention was paid to the SZ's state of arousal, which was constantly monitored by a camera inside the shielded room during imaging, and we confirmed that the SZ's arousal state was adequately maintained during imaging. After the measurements, the Stanford Sleepiness Scale was used to check participants’ wakefulness.

***The Stanford Sleepiness Scale***

After the measurements, in addition to the video recording, the Stanford Sleepiness Scale was used to confirm participants’ wakefulness. We did not detect a significant difference in wakefulness between the SZ group and the HC group (median, interquartile range:3:0, 2.0-4.0, vs. 3.0, 3.0-4.0, z-score = -0.386, p > 0.05). This indicates that the degree of arousal states in both groups remained adequate during the measurement, and that the results of the analysis associated with the group differences were not explained by the degree of participants’ wakefulness.

**Preprocessing**

As preprocessing to remove system noise such as alternating current (AC) and biogenic artifacts such as heartbeat and blinking from the acquired MEG signal, we first applied Max filter 2.0 (Elekta-Neuromag) which is a spatial filter recommended by the manufacturer to separate actual brain activity from the other unrelated signals. Second, signal space projection was used to remove biogenic noise identified in the EOG and ECG measurements. Third, to remove biological noise other than EOG and ECG (e.g., artifacts due to body motion), an independent component analysis was performed, and components with amplitudes greater than 2000 fT were removed if they existed in the data. Subsequently, the power spectrum density was calculated to investigate the effect of the AC current. Based on the calculation results, 50 Hz and its harmonic components (100 Hz, 150 Hz, 200 Hz, 250 Hz, 300 Hz, 350 Hz, 400 Hz, 450 Hz, and 500 Hz) were removed up to a Nyquist frequency (500 Hz) using a notch filter. The sampling rate was then downsampled from 1002 to 1000 Hz.

**T1-weighted MRI scanning**

We acquired structural MRI T1-weighted images from all participants using a 3T MRI scanner (Siemens, Erlangen, Munich, Germany) with a 12-channel phase-array receiver coil at Gunma University Hospital. A voxel size of 1 $\times$ 1 $\times$1 mm was acquired using magnetization-prepared rapid acquisition with a gradient-echo sequence. The imaging parameters were as follows: repetition time (TR) = 2000 ms; echo time (TE) = 2 ms; inversion time = 990 ms; flip angle = 9 °; acceleration factor, 3D = 1; field of view, 256 × 256 mm; matrix size, 256 × 256.

We also used a 3T MRI device with a 12-channel phase-array receiver coil at Josai Clinic (Philips Medical Systems, Best, Netherlands). The parameters for imaging were set as followings: TR = 2000 ms; TE = 2.8 ms; flip angle = 9°; field of view = 256 × 256 × 211 mm (anterior to posterior × foot to head × right to left); matrix size, 256 × 256; and voxel size, 1 × 1 × 1 mm.

**Issues of auto-detection of SPW-Rs with MEG**

Recent years have seen increased interest in SPW-R detected with MEG research in humans^3,4^, posing challenges related to their detection criteria. In rodent studies, SPW-Rs are typically identified using a specific frequency band (e.g., 100-250 Hz for rodents, 70-250 Hz for humans) and an amplitude threshold (e.g., 2-7 standard errors) based on background activity of local field potentials measured with a probe implanted in the CA1 pyramidal layer of the hippocampus. Additional confirmation methods in rodents include spike analysis, layer analysis, neuron type identification (e.g., chandelier and O-LM interneuron), and responses to inputs from cholinergic neurons. However, applying rodent detection protocols, like installing sEEG into the CA1 pyramidal layer, is challenging in humans. Notably, the standard for noninvasive measurement of SPW-Rs, such as MEG, is not well established.

While our current protocol is validated and fine-tuned through simultaneous measurements via sEEG and MEG, we emphasize the importance of physiologically valid event thresholding (Figure 1B) and PAC analysis (Figure 3) for SPW-Rs validation. These neurophysiological properties play a crucial role in distinguishing SPW-Rs from high-frequency oscillations (e.g., high gamma 50-120 Hz). For example, SPW-Rs exhibit specific physiological characteristics, such as a long-tailed distribution (gamma or log-normal distribution) in duration, while high gamma oscillations show more sustained activities. Additionally, the amplitude of the high gamma band is phase-locked to theta oscillations, whereas SPW-Rs are phase-locked to spindle activity. Both these properties contribute to differentiating SPW-Rs from high gamma oscillations. Although our study ensures the reliability of the detected events by evaluating these properties, refining a more reliable protocol for human SPW-Rs detection remains a priority.

**The kerel design of the Complex Morlet wavelets (CMW)**

CMW is a typically designed with the parameter “number of cycles”, which relates to the combination of complex sine waves and a Gaussian window. However, the use of the number of cycles can introduce opacity and uncertainty in the analysis results. In this regard, using "the full width at half maximum (FWHM)" as an alternative is known to provide clearer and stable analysis results^1^. In this study, we employ FWHM for the kernel design of Continuous Morlet Wavelets (CMW). For the kernel design of the complex Morlet wavelets, two parameters were predetermined: the central frequency and the full width at half maximum (FWHM) for the Gaussian function. The former was defined as 40 linearly spaced steps between 50 and 250 Hz. The full width at half maximum (FWHM) was set to vary linearly from 1000 to 200 ms with increasing central frequency.

**Phase amplitude coupling (PAC) via Wasserstein Modulation Index (wMI)**

**
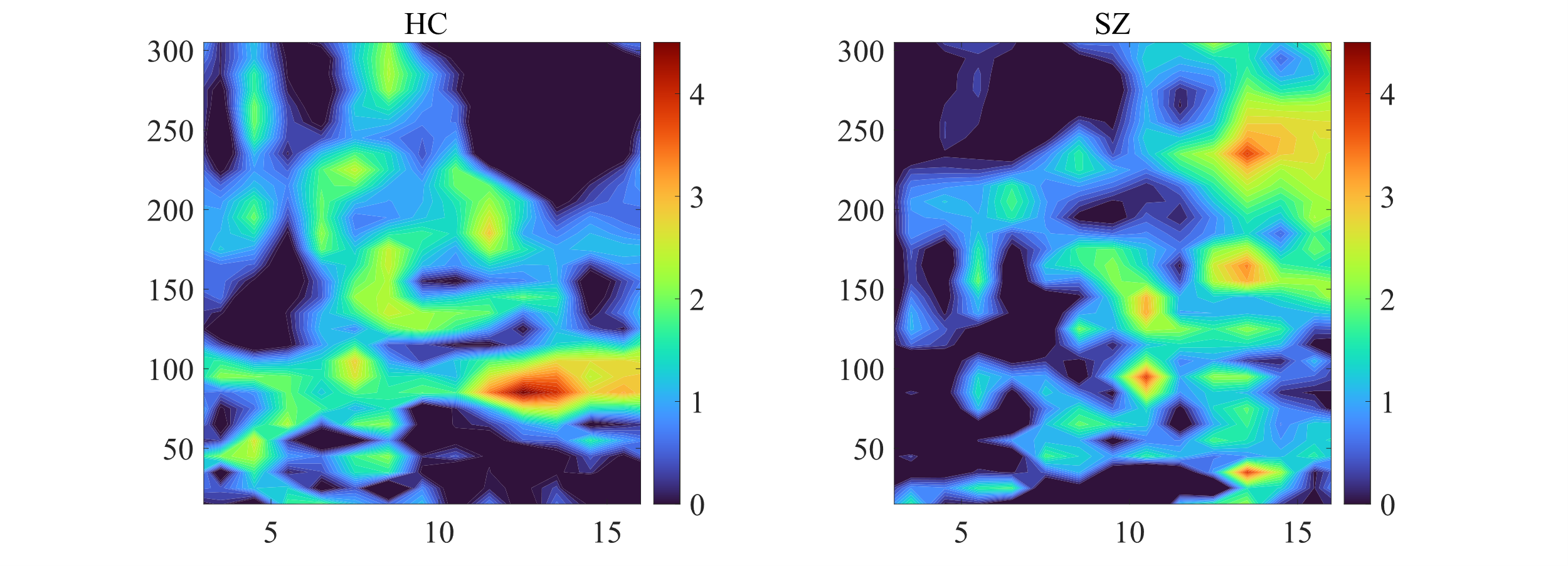
**

Figure S1. Comodulograms without thresholding.

In this figure, we present the analysis results of raw PAC without p-value or cluster-size correction. The left and right panels depict the results for the control group and SZ group, respectively. Regardless of threshold application, it is important to note that in the HC group, PAC patterns were detected as clusters, while in the SZ group, more fragmented PAC patterns were observed.

**Ripple-gedMEG and Shrinkage regularization (SR)**

One of the main advantages of the current study was the quantification of SPW-Rs using whole-brain measurements from multiple MEG sensors. To fully exploit this advantage, we used a novel approach to analyze SPW-Rs via generalized eigendecomposition (GED), which allowed us to reveal the whole brain network consisting of SPW-Rs. For this purpose, we applied the GED. Mathematically, the GED calculation automatically finds eigenvectors that maximize the difference between two multidimensional datasets tailored to the research objectives. And, an eigenvalue is a scalar that reflects the length of the eigenvector, which reflects how well the source separation via GED performs (in other words, a signal-to-noise ratio (SNR)). It's important to note that when the eigenvalue is equal to 1, it indicates that the source separation does not work well.

In the GED calculation, we first created two covariance matrices (i.e., the target covariance matrix [channel-by-channel] consisted of SPW-Rs (80-250 Hz) and the control covariance matrix consisting of the raw brain signals [channel-by-channel] (Figure 4A and S2). These two covariance matrices were used to perform eigenvalue decomposition. Specifically, GED can be expressed as

*CWΛ=TW*

or

*WΛ = (C^-1^T)W*

where *C* and *T* denote the control and target covariance matrices, respectively. Thus, the GED can be intuitively understood to find eigenvectors (*W*) denoting channel weights associated SPW-Rs, and *Λ* representing SNR. Importantly, we applied regularization to *C*. Regularization adds a constant to the cost function of an optimization algorithm, providing the benefit of smoothing the solution to reduce overfitting and increase numerical stability, especially for low-rank datasets (Figure S2). Among several regularization methods such as Lasso, Ridge and others, we used shrinkage regularization (SR), since SR is simple and proved to be effective in GED^2^. Especially in the reconstruction of weak spontaneous brain oscillations, such as ripple, SR is highly effective. The mathematical formulation of SR is as follows:

$$\tilde{C}=C\left( 1-\gamma\right)+ \gamma\alpha I_{N}$$

$$\alpha= \sum_{i=1}^{N} \lambda_{i}$$

$\tilde{C}$, $\gamma$, $\alpha$and $I_{N}$denote the regularized control covariance matrix, the regularization amount, the average of all eigenvalues of $C$ and the $N \times N$ identity matrix (i.e., channel by channel) respectively. The calculation of SR requires a pre-set value for $\gamma$, which in the current study was set to 0.01 (i.e., 1% regularization). Incidentally, we named this novel approach ripple-gedMEG because the design of the covariance matrices was specific for SPW-Rs combined with MEG measurements.

Note that eigenvectors have fundamental sign uncertainty and potentially affect the signs of topographical maps. Accordingly, if the largest magnitude of the eigenvectors has a negative value, the entire eigenvector was multiplied by -1 (this flipping sign is often used in the principal component analysis).


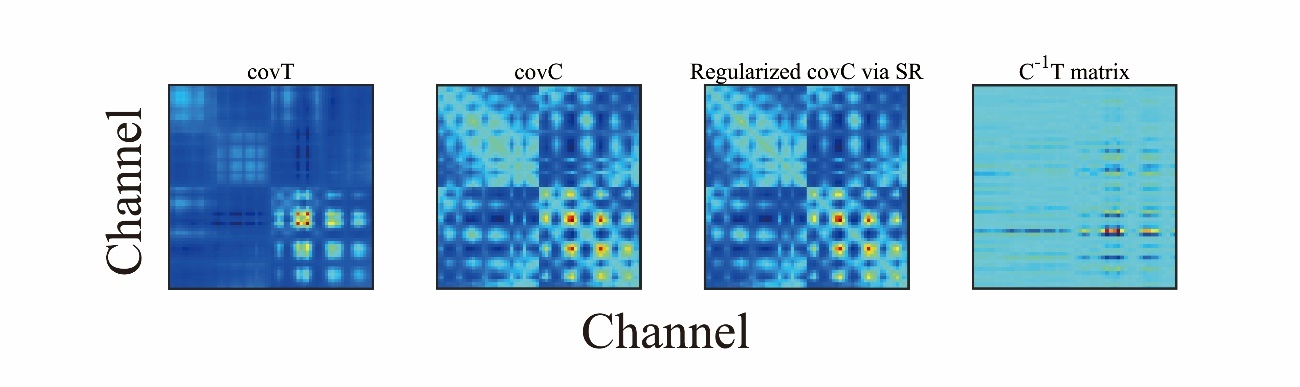


Figure S2. Examples of four covariance matrices.

From left to right: the target covariance data (covT) reflecting the spatial distribution of SPW-Rs, Control covariance data generated by raw data (covC), covC after regularization, and the C^-1^T matrix reflecting the results of GED calculation. Note these results were created from the simulated SPW-Rs data.

**Simulation Code for validity and superiority of ripple-gedMEG**

We validated the ripple-gedMEG using simulation data to demonstrate its robustness and superiority, based on previous reserach^2^. I. In our simulation, we generated a single ripple source (120 Hz), a phase-amplitude coupled signal (with 15 Hz for phase and 120 Hz for amplitude, representing nesting and nested frequencies, respectively), and a gaussian noise at thousands of dipole locations in the brain, mirroring our empirical findings (Figure 1 and 2). The target signal had a 1-second duration with a sampling rate of 1 kHz. We specified the starting time and duration of events at 500 ms and 50 ms, respectively (see Figure S3). The time series data passed through a leadfield matrix to simulate the signal in the sensor space. For comparison, principal component analysis (PCA, Figure S4) and independent component analysis (ICA, Figure S5) were used because these two algorithms are similar dimensionality reduction methods with GED^2^. The sensor-level brain activity from this simulation was then analyzed using PCA, ICA, and ripple-gedMEG (Figure S6). In conclusion, ripple-GED has been demonstrated to surpass both PCA and ICA in terms of source separation and the reconstruction of temporal patterns. Notice that the basic concept for the design of our simulation is based on the following paper. The core methodology of this research will be downloaded from the website (<https://github.com/Ohki3139/ripple-gedMEG>).


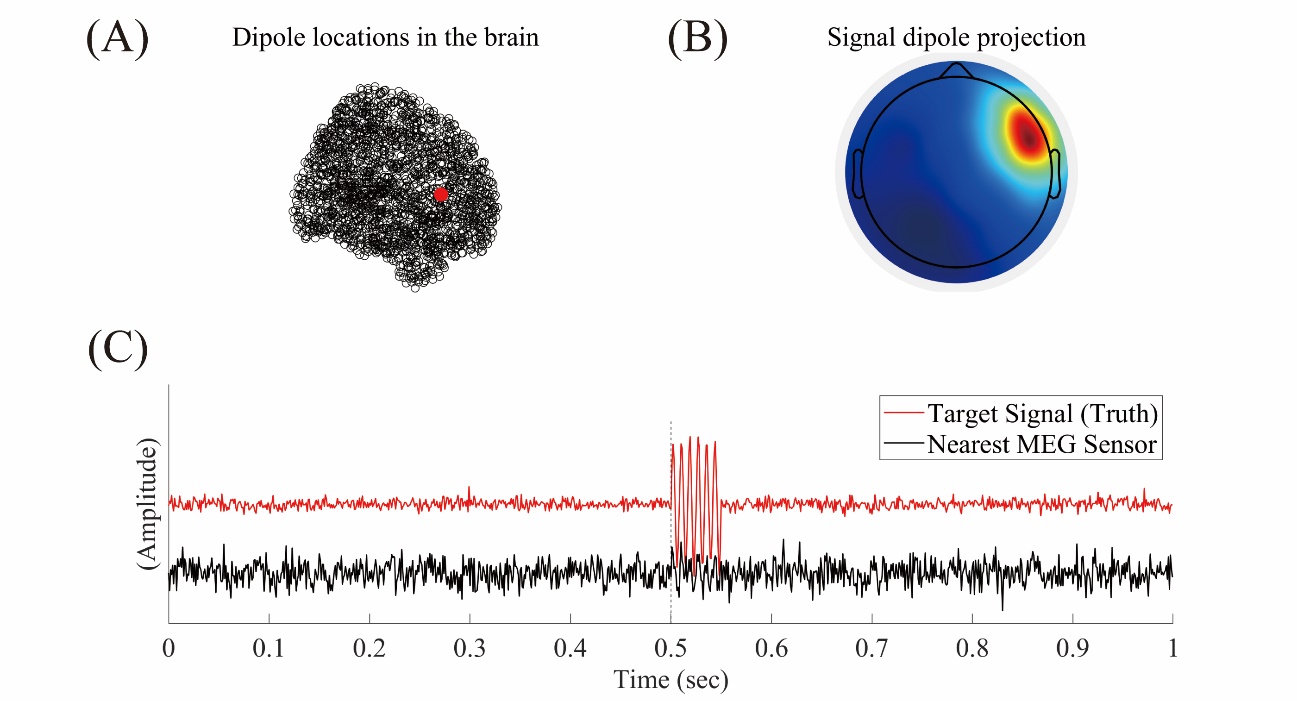


Figure S3. The simulation for reconstruction of SPW-Rs.

(A) Target dipole locations associated with SPW-Rs in the brain. Each black and single red dot denote dipole locations showing time series consisting a gaussian noise and the target signal.

(B) The brain source projection in the sensor space. The red color denotes the sensor positions reflects the target signal.

(C) The red and black time series denote the target time-series data as the ground truth and the target time series data detected in the nearest sensor to the target signal source respectively. Note that when a single source is projected onto the sensor space, a single sensor may not accurately reproduce the temporal sequence of the ground truth data.


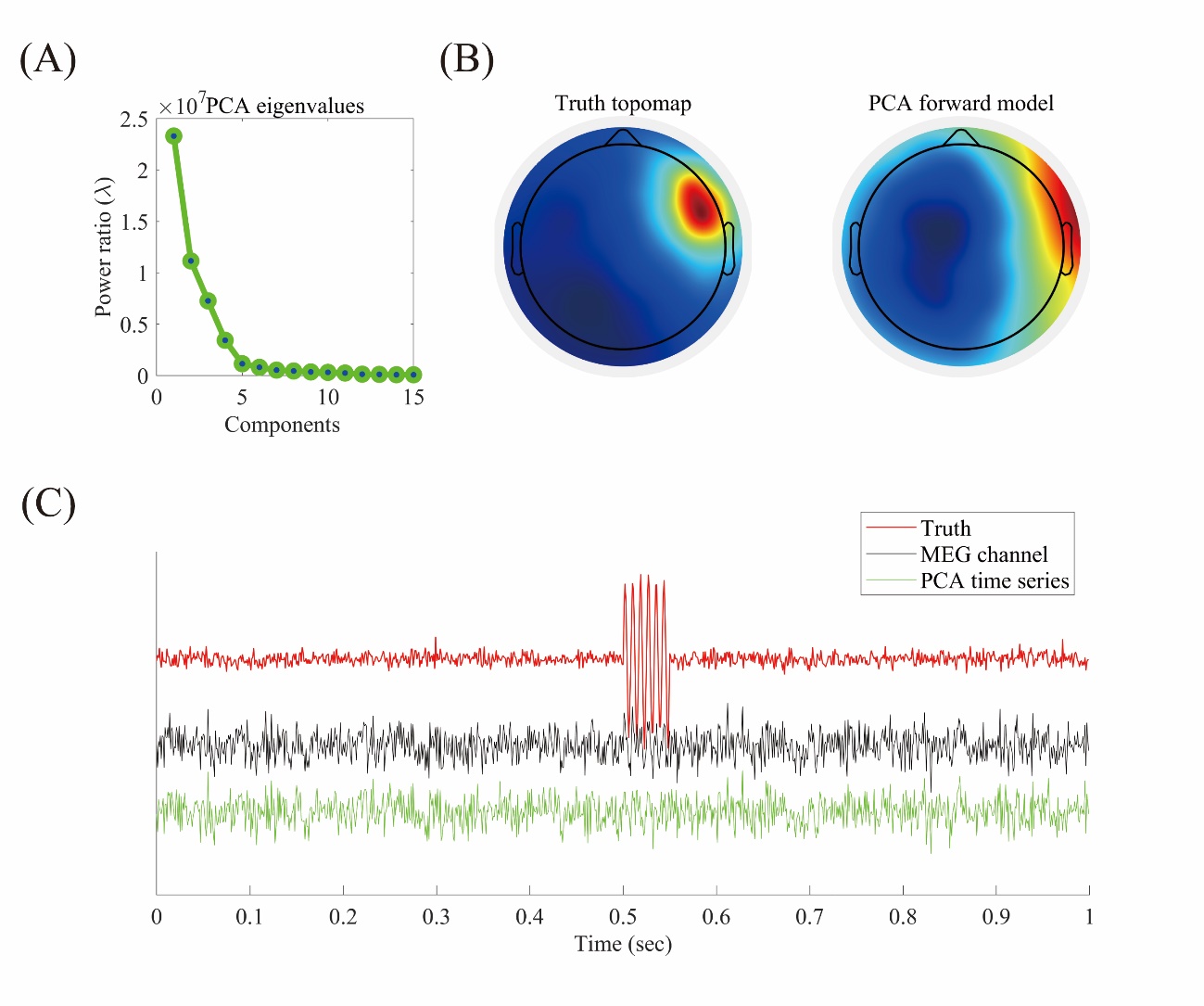


Figure S4. Reconstruction of the target signal via PCA.

(A) Eigenvalues of PCA.

(B) The target dipole projection in the sensor space as the ground truth and source reconstruction via PCA (i.e., the forward model).

(C) The green time series data represents the time series waveform (i.e., the 1st principal component) restored by PCA. It is evident that PCA failed to restore the oscillatory nature of the target data. The red and black time series data are identical to Figure S3.


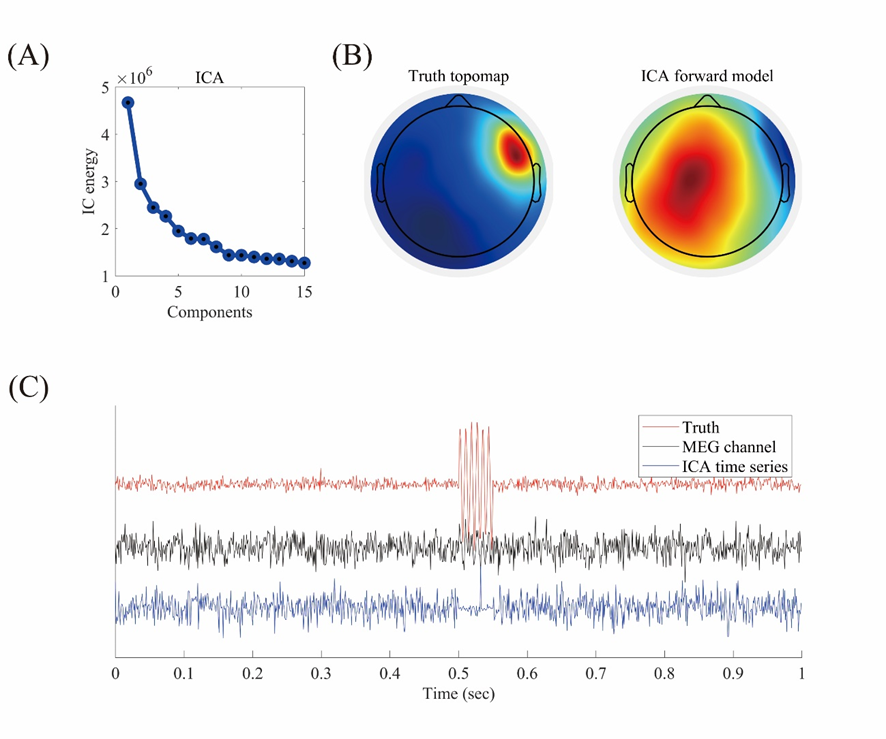


Figure S5. Reconstruction of the target signal via ICA.

(A) Energy of ICs.

(B) The target dipole projection in the sensor space as the ground truth and source reconstruction via ICA.

(C) The blue time series data denote the 1st independent component. It became clear that ICA also failed to restore the oscillatory nature of the target data. The red and black time series data are identical to Figure S3.

**
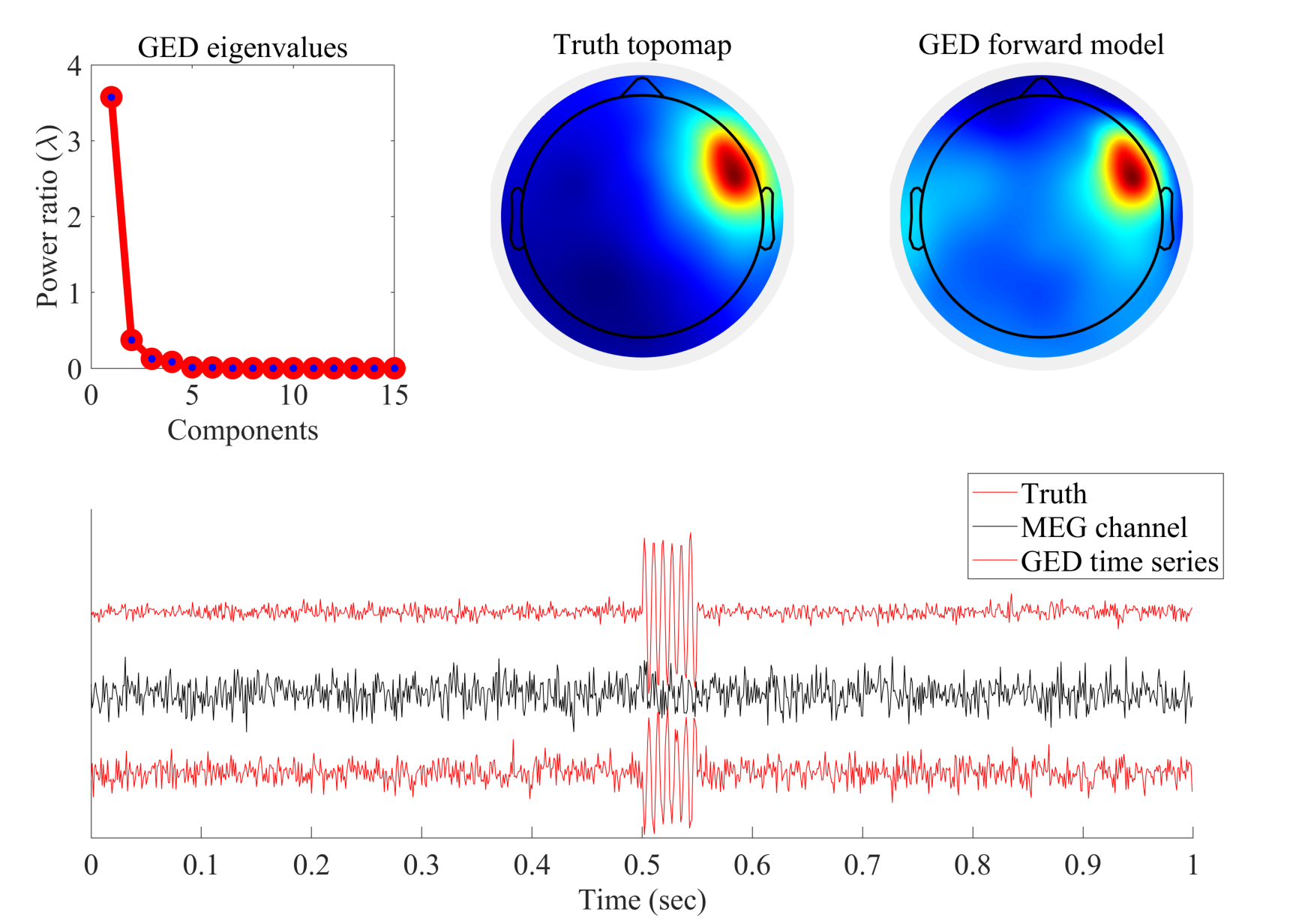
**

Figure S6. Reconstruction of the target signal via GED.

(A) Eigenspectrums of GED.

(B) The target dipole projection in the sensor space as the ground truth and source reconstruction via GED.

(C) The red time series data represents the 1st component. It is evident that the oscillatory nature of the target data could be fully restored via GED. The red and black time series data are identical to Figure S3.

**Statistical evaluations via permutation tests**

*The number of events*

To investigate the differences in the number and duration of events between the groups, we performed a permutation test. In this process, we allocated group indices, such as 0 for the HC and 1 for the SZ, and randomly shuffled both sets of data via these indices 1000 times (i.e., H0 distribution of the number of SPW-Rs in green shown in Figure 1C right). Using this H0 distribution, we determined the significant differences in the number and duration of events between the two groups.

*Phase amplitude coupling*

First, we created surrogate data by swapping the time series back and forth 1000 times at random time points. We used this surrogate data to statistical evaluate whether the observed wMI values and coupling phase matrices were significant (z = ± 3.29, p < 0.001, Figure 3AD). After creating the surrogate data, the observed wMI and coupling phase matrix were converted to z-scores, and p-values were obtained. The second surrogate dataset was generated primarily for group comparison purposes, denoted as indirect and direct comparisons in Figure 3C. In the indirect comparison, we used the second surrogate dataset created by assigning group labels to the wMI values and randomly shuffling these labels 1000 times. We examined the significant differences of the wMI values between HC and SZ for the second null hypothesis distribution (z = ± 3.29, p < 0.001).

Another unique computational property of wMI is that, by setting the constraint terms (reference = NC, target = SZ) appropriately, it is possible to directly compare the PAC distribution between the two groups, as shown in Figure 3B (i.e., direct comparison). Therefore, we performed a direct group comparison between the HC and SZ groups using wMI. To create the third surrogate dataset for direct group comparison (right panel in Figure 3C), the PAC distributions were randomly shuffled 1000 times based on the group labels (i.e., HC = 0, SZ = 1). The surrogate data was used to calculate z and p values to determine significance for the wMI values and the coupling phase matrices (z = ± 3.29, p < 0.001). For some figures (Figure 3A and D), a threshold based on p-values (z = ± 3.29, p < 0.001) was set just for a simple visualization purpose, and values that did not reach the threshold were replaced with 0.

**Correction for multiple comparisons using cluster-based statistics**

We employed cluster-based statistics (cluster-size correction)^5^ as a multiple comparison correction for the PAC map (comodulogram demonstrated in Fig. 3A right panels). This method applies a null hypothesis distribution (H0) of cluster sizes obtained from surrogate data to significant pixels on the PAC Map (Fig 3A left panels, p < 0.01, permutation test). In this approach, we firstly used the built-in MATLAB function bwconncomp to detect significant clusters in the surrogate data and calculate their maximum size. This process was repeated 1000 times for each of the 1000 surrogate data, allowing us to use these data as H0 for cluster sizes. These H0 cluster size threshold (p < 0.01) was applied to the original PAC z-score map and non-significant clusters were replaced with 0 (the right panels Fig. 3A).

*Ripple-gedMEG*

As a statistical process in ripple-gedMEG, we first compared the median eigenvalues, gamma power, and SPW-R power obtained for each participant between groups using the Wilcoxon rank-sum test and permutation test by shuffling the group labels (e.g., HC = 0, SZ = 1) 1000 times. Notably, we have already verified the superiority and robustness of ripple-gedMEGs using various simulated and empirical data (Ohki, in press).

*The brain state transitions*

To determine the statistical significance of the brain state transition probability changes associated with the occurrence of SPW-Rs (Figure 5G), we used permutation by shuffling the condition labels (SPW-Rs = 0 or 1) 1000 times in both groups. To further clarify the group differences in transition probabilities, we created two subtraction matrices of the transition probabilities (i.e., HC_1_-SZ_1_ and SZ_1_-HC_1_) for visualization purposes and performed a permutation test by randomly shuffling group labels (i.e., HC_1_ = 0 and SZ_1_ = 1) 1000 times.

**Reference**

1. Cohen MX. A better way to define and describe Morlet wavelets for time-frequency analysis. *Neuroimage*. 2019; **199**: 81-86.

2. Cohen MX. A tutorial on generalized eigende composition for source separation in multichannel electrophysiology. *Neuroimage*. 2022; **247**: 118809.

3. Ruzich E, Crespo-García M, Dalal SS, Schneiderman JF. Characterizing hippocampal dynamics with MEG: A systematic review and evidence-based guidelines. *Hum Brain Mapp*. 2019; **40**: 1353-1375.

4. Pizzo F, Roehri N, Medina Villalon S *et al*. Deep brain activities can be detected with magnetoencephalography. *Nat Commun*. 2019; **10**: 971.

5. Cohen MX. Analyzing neural time series data: theory and practice. MIT press, 2014.
